# International Lower Limb Collaborative (INTELLECT) study: a multicentre, international retrospective audit of lower extremity open fractures^[Author-notes znac105-FM2]^

**DOI:** 10.1093/bjs/znac105

**Published:** 2022-04-26

**Authors:** Juan Enrique Berner, James K K Chan, Matthew D Gardiner, Alfonso Navia, Rodrigo Tejos, Manuel Ortiz-Llorens, Alina Ortega-Briones, Hinne A Rakhorst, Jagdeep Nanchahal, Abhilash Jain, INTELLECT Collaborative, G Nolan, H Samarendra, A Mohan, K Cooper, N Pereira, G Mangelsdorff, J Venegas, A Layseca, J Skillman, A Kennedy, A Qureshi, K Wallis, L Harry, A Hagiga, S Ibrahim, M Albendary, K A Shah, C B Chuo, C Katsura, J R Rodríguez Astudillo, A López Ortega, J P Henríquez Rissios, M Nova Nova, J Hughes, C Wearn, D Peberdy, B Ho, K Gohil, A Abood, N Rabey, M Nizamoglu, G Biosse-Duplan, K To, S R Sabapathy, M Mohan, H Venkatramani, S Rajasekaran, H Hsu, A R Ambriz Plascencia, L E Escalona Ramírez, C A Zepeda Torres, E Santamaria, S Vallejo Toro, C West, W Bhat, C McArdle, S Louette, S Hassan, P W van Egmond, W J J Bekkers, D Capitani, L Troisi, T Talamonti, P Capitani, V Cerbone, G Materazzi, L Ballini, J Tomas-Hernandez, J A Porcel-Vazquez, Y Garcia-Sanchez, J V Andrés-Peiró, J Teixidor-Serra, J Selga-Marsà, H Dafydd, S Ali, R Slade, S Tarassoli, B Olías López, J Boluda Mengod, D González Martín, A Bashir, A Dearden, V Itte, F Smith, W C Lee, V A A Paulus, P Romijn, T N Tromp, T de Jong, S Koide, K Lim, F Raiola, S Ferris, A Rodríguez, E L Jonsson, S Holm, O Wolff, A Abugarja, H Elbahari, H K S Hamid, M Awadelkarim, J Erdocia Pascual, L Bahillo O'Mahoney, M A Quiroga Bilbao, M Felipe Peña, W Eardley, A Egglestone, S Taher, N Wei, J Martínez Ros, G Valero Cifuentes, A Ondoño Navarro, A Escudero Martínez, A Ortega Columbrans, P Zamora, J Masiá, A Ibarra, M Fernández, V Giblin, A Kilshaw, B Wood, M Wyman, I E Tinhofer, E Seidl, C J Tzou, S Quadlbauer, J Reichetseder, H Bürger, T Hausner, S van Miltenburg, I Beijk, W Verra, R de Groot, V Kunc, L Kopp, A Crick, C Mitchell, T Curran, R Kuo, S Eltoum Elamin, P Caba Doussoux, D Alonso Tejero, J Gómez Alcaraz, J M Pardo García, K Kooi, R Poelstra, J P Hong, M Jang, D W Hong, J G Kwon, M Francés Monasterio, J Fernández-Palacios Martínez, A Suarez Cabañas, M Marrero Martínez-Carlón, W ten Cate, J E D Jacobs, J Rawlins, J Haley, J Palma, A Cuadra, H Demandes, S Canahuate, D Moreno, S Norton, J Thompson, G Lafford, D Noriego Muñoz, A Teixido de la Cruz, M Vázquez Gómez, A Mingoli, D Ribuffo, G Marruzzo, P Lapolla, W Ayad, A Elbatawy, M Ouf, P Castillón, C García, M Surroca, J Garcia-Coiradas, F Marco, M Cherubino, L Garutti, G Molina Olivella, A Endemaño Lucio, K Oflazoglu, F Lutgendorff, M Botman, G Giannakopoulos, R Dams, P van der Zwaal, R Moral-Nestares, F Requena, N Fernández-Poch, J Cámara-Cabrera, F Macán, M Standen, F Flaherty, M Vizcay, D Yerson, E Sperone, F Bidolegui, S Pereyra, E Chouhy, M Jaureguialzo, Z Arnez, V Cazzato, M A Giraldez, R Moreno Domínguez, B Martínez Sañudo, L Lancerotto, S Sandhu, A Robinson, C Digney

**Affiliations:** Department of Plastic Surgery, Royal London Hospital, London, UK; Kellogg College, University of Oxford, Oxford, UK; Department of Plastic Surgery, Stoke Mandeville Hospital, Aylesbury, UK; Nuffield Department of Orthopaedics, Rheumatology and Musculoskeletal Sciences, University of Oxford, Oxford, UK; Department of Plastic and Reconstructive Surgery, Wexham Park Hospital, Slough, UK; Kennedy Institute of Rheumatology, Nuffield Department of Orthopaedics, Rheumatology and Musculoskeletal Sciences, University of Oxford, Oxford, UK; Sección de Cirugía Plástica y Reconstructiva, División de Cirugía. Facultad de Medicina, Pontificia Universidad Católica de Chile, Santiago, Chile; Sección de Cirugía Plástica y Reconstructiva, División de Cirugía. Facultad de Medicina, Pontificia Universidad Católica de Chile, Santiago, Chile; Facultad de Ciencias, Universidad Mayor, Santiago, Chile; Trauma and Orthopaedic Surgery Department, Hospital San José Quirónsalud, Madrid, Spain; Plastic and Reconstructive Surgery Department, Medisch Spectrum Twente, Enschede, the Netherlands; Kennedy Institute of Rheumatology, Nuffield Department of Orthopaedics, Rheumatology and Musculoskeletal Sciences, University of Oxford, Oxford, UK; Nuffield Department of Orthopaedics, Rheumatology and Musculoskeletal Sciences, University of Oxford, Oxford, UK

## Abstract

Sixty-two centres in 16 countries contributed with 2,694 open fractures cases to an international, multi-centric, retrospective cohort study involving different healthcare settings. The INTELLECT study results show that there are significant disparities on the management of open lower limb fractures internationally. A timely, multidisciplinary, guideline-directed care is a protective factor for developing infective complications, non-union and requiring an amputation.

## Introduction

Trauma remains a major cause of mortality and disability across the world^1^, with a higher burden in developing nations^2^. Open lower extremity injuries are devastating events from a physical^3^, mental health^4^, and socioeconomic^5^ standpoint. The potential sequelae, including risk of chronic infection and amputation, can lead to delayed recovery and major disability^6^. This international study aimed to describe global disparities, timely intervention, guideline-directed care, and economic aspects of open lower limb injuries.

## Methods

The INTELLECT (International Lower Limb Collaborative) study was an international, multicentre, retrospective audit supported by the Reconstructive Surgery Trials Network. Investigators in participating centres were tasked to retrieve demographic and clinical data for patients who had an open lower extremity fracture treated between 1 January 2017 and 31 December 2018. Primary outcomes were soft tissue infection, deep infection, non-union, and amputation. Secondary outcomes were median time to discharge and instances of deep venous thrombosis. Details of the study protocol and inclusion criteria can be found in *Appendix S1* and *Table S1* respectively. According to National Health Service Health Research Authority guidance, this study was not considered to be research, but an audit. Therefore, no formal ethics approval was required for centres in the UK. For other countries, local policies regarding information governance were adhered to.

## Results

Sixty-two centres in 16 countries contributed with 2694 patients, a median of 17 per unit each year. Mean age at presentation was 44.5 (range 2–100) years, and 104 patients (3.9 per cent) were aged less than 16 years. Some 71 per cent of the affected individuals identified as male, and median follow-up was 11 (range 0–47) months (*Fig. S1*). The most common mechanism of injury was road traffic accidents (52.6 per cent), followed by low-energy (18.7 per cent) and high-energy (11 per cent) falls (*Table S2*). A descriptive summary, including demographic information, treatment provided and outcomes, can be found in *Tables 1* and *2*.

**Table 1:** Descriptive baseline information uploaded to INTELLECT database

	Total (*n* = 2694)	Femoral fractures (*n* = 272)	Tibial/fibular fractures (*n* = 2131)	Hindfoot fractures (*n* = 291)
Age (years)* **(*n*** = **2670)**	44.5(20.4)	37.9(17.9)	45.5(20.8)	43.5(19.1)
**Gender ratio (M : F) (*n*** = **2690)**	71 : 29	79 : 21	69 : 31	72 : 28
**Mechanism of injury (%) (*n*** = **2656)**				
Road traffic accident	53	70	52	37
Low-energy fall	19	9	11	13
High-energy fall	11	4	21	17
Sports-related	6	1	4	12
Work-related	4	2	6	8
Interpersonal violence	5	12	5	5
Other	2	2	1	8
**Fracture classification (Gustilo–Anderson) (%) (*n*** = **2593)**				
I	16	16	15	21
II	31	38	29	38
IIIA	20	31	19	15
IIIB	28	9	32	21
IIIC	5	6	5	5
**No. of patients per country (*n*** = **2694)**				
UK	1045	73	918	54
Spain	396	51	288	57
Chile	322	35	216	71
Netherlands	227	30	180	27
Mexico	113	14	86	13
Italy	98	18	74	6
Austria	78	5	60	13
Australia	70	2	53	15
India	60	6	54	0
Taiwan	59	14	38	7
Argentina	50	2	48	0
Sweden	45	6	24	15
Sudan	43	7	29	7
Czechia	37	9	25	3
South Korea	31	0	23	8
Egypt	20	0	15	5
**Co-morbidities (%) (*n*** = **2447)**				
None	65	73	63	70
Hypertension	16	9	17	15
Diabetes	8	7	9	6
Ischaemic heart disease	4	4	4	2
Asthma	4	2	4	5
COPD	3	2	3	3
Peripheral vascular disease	1	1	2	1
**Smoker (%) (*n*** = **2666)**				
Yes	24	26	23	25
No	42	32	43	43
Unknown	34	42	34	32

*Values are mean(s.d.). COPD, chronic obstructive pulmonary disease. Reported missing values: 2.7 per cent.

**Table 2:** Treatment and outcomes data uploaded to INTELLECT database

	Total (*n* = 2694)	Femoral fractures (*n* = 272)	Tibial/fibular fractures (*n* = 2131)	Hindfoot fractures (*n* = 291)
Time to antibiotics (h)* **(*n*** = **2303)**	2 (1–5)	2 (1–4)	2 (1–5)	2 (1–5)
**Direct transfer to specialist centre (%) (*n*** = **2670)**	75	83	73	78
**Wound debridement within 24 h (%) (*n*** = **2580)**	80.2	86.3	79.6	79
Time to debridement (h)*	10 (4–20)	6.5 (3–15)	10 (5–21)	8 (4–20)
**Specialties involved in primary debridement (%) (*n*** = **2694)**				
Orthopaedic surgeons	68	80	65	79
Plastic surgeons	11	4	12	6
Orthopaedic and plastic surgeons	14	5	16	11
Trauma surgeons	7	10	7	4
**Seniority of surgeon leading debridement (%) (*n*** = **2619)**				
Consultant level	80	84	80	81
Non-consultant level	20	16	20	19
Median time to definitive skeletal fixation (days)* **(*n*** = **2670)**	2 (1–9)	4 (1–9.5)	2 (1–9)	1 (0–5)
**Primary mode of definitive skeletal fixation (%) (*n*** = **2670)**				
Casting	4	1	3	14
Uni/biplanar external fixator	7	2	7	6
Frame external fixator	12	5	14	4
Plate and screws	33	29	33	38
Intramedullary nail	36	58	38	2
Kirschner wires	4	1	2	27
Other	4	4	3	9
**Soft tissue reconstruction required (%) (*n*** = **2672)**	41	18	44	41
**Modality of soft tissue closure (%) (*n*** = **1109)**				
Conventional dressings	6	8	6	9
Negative pressure wound therapy	5	6	4	11
Skin grafting only	21	56	20	19
Local flaps	9	4	10	3
Perforator flaps	16	11	17	3
Free flaps	43	15	43	55
Time to soft tissue closure (days)* **(*n*** = **1061)**	7 (3–19)	12 (4–22)	6 (3–17)	16 (4–28)
**Flap survival rate (%) (*n*** = **748)**				
Local and regional flaps	(*n* = 268)	(*n* = 7)	(*n* = 255)	(*n* = 6)
Total flap failure	3	14	3	0
Partial flap failure	9	0	9	0
Total flap survival	88	86	88	100
Free flaps	(*n* = 480)	(*n* = 7)	(*n* = 408)	(*n* = 65)
Total flap failure	6	0	7	2
Partial flap failure	6	29	5	9
Total flap survival	87	71	88	89
**Deep venous thrombosis (*n*** = **2694)**	35 (1.3)	4 (1.5)	27 (1.3)	4 (1.4)
**Wound infection (%) (*n*** = **2649)**	16.3	12.5	16.7	16.9
Time to wound infection (days)*	34 (13–80)	15 (7–41)	39 (16–90)	17 (8–37)
**Deep wound infection (%) (*n*** = **2655)**	10.3	9.4	10.7	8.6
Time to deep infection (days)*	63 (23–180)	52 (19–162)	69 (27–184)	32 (14–63)
**Non-union (%) (*n*** = **2594)**	10.9	14.7	11.7	2.1
**Amputation (%) (*n*** = **2624)**				
Immediate	2.1	1.9	1.4	7
Early	2.7	0.8	2.3	7.4
Late	1	0.8	1.1	0
Time to discharge (days)* **(*n*** = **2641)**	15 (7–30)	16 (8–33)	15 (7–29)	14 (4–30)
Median follow-up (months)* **(*n*** = **2448)**	11 (4–20)	13 (5–22)	11 (4–20)	10 (2–19)

Values in parentheses are percentages unless indicated otherwise; *values are median (i.q.r.). Reported missing values: 2.7 per cent.

A multivariate logistic regression analysis adjusting for age, mechanism of injury, and country income group showed that patients with Gustilo IIIB and IIIC fractures had a higher likelihood of wound infection (odds ratio (OR) 1.44; *P* = 0.047) or secondary amputation (OR 2.39; *P* = 0.027). There was no significant statistical association between time to antibiotics, time to debridement, or time to definitive fixation with soft tissue infection, deep infection, non-union, and amputation. However, male gender identity was a risk for developing superficial wound infection (OR 1.5; *P* = 0.038), deep infection (OR 1.68; *P* = 0.035), non-union (OR 1.96; *P* = 0.016), and amputation (OR 8.02; *P* = 0.004). Delay in achieving soft tissue closure beyond 72 h after injury was associated with a greater likelihood of developing soft tissue infection, deep infection, non-union, and requiring an amputation (*Table S3*). Joint treatment by orthopaedic or trauma surgeons working with plastic surgeons from the time of the debridement onwards was identified as protective factor for secondary amputation (OR 0.41; *P* = 0.008).

Two-thirds of injuries that required soft tissue reconstruction were previously classified as Gustilo IIIB and IIIC, 76 per cent in high-income countries with guidelines, 58 per cent in high-income countries with no guidelines, and 63 per cent in low- and middle-income countries. There were differences in surgical-site infections and duration of follow-up (*Table 3*). Multivariable analysis showed that being treated in a setting with national guidelines^7–9^ was protective with respect to developing a deep tissue infection (OR 0.66; *P* = 0.040) or non-union (OR 0.66; *P* = 0.043), with a 34 per cent lower likelihood of developing either of these.

**Table 3:** Comparison of management and outcomes following open tibial/fibular fractures in high-income countries with and without national guidelines and middle- and low-income countries

	High-income countries with guidelines (*n* = 1098)	High-income countries with no guidelines (*n* = 801)	Middle- and low-income countries with no guidelines (*n* = 232)	*P*‡
% Gustilo IIIB–IIIC (*n* = 2113)	45.3	26.2	35.5	< 0.001
Age (years)* (*n* = 2113)	48.1.(22.1)	45.2.(18.5)	34.1.(17.3)	< 0.001
Gender ratio (M : F) (*n* = 2127)	63 : 37	74 : 26	80 : 20	< 0.001
Time to antibiotics (h)† (*n* = 1823)	2 (3)	2 (3)	5 (8)	< 0.001
Time to debridement (h)† (*n* = 2044)	14 (16)	6 (11)	11 (17)	< 0.001
Time to fixation (days)† (*n* = 2044)	2 (6)	3 (12)	5 (12)	< 0.001
Time to soft tissue cover (when required) (days)† (*n* = 2044)	4 (7)	12 (23)	8 (35)	< 0.001
Multidisciplinary debridement (%) (*n* = 2131)	24.4	7.0	6.9	< 0.001
Consultant-led debridement (%) (*n* = 2073)	77.4	90.7	56.9	< 0.001
Wound infection (%) (*n* = 2095)	17.7	14.2	20.3	0.040
Deep infection (%) (*n* = 2100)	8.8	11.6	15.9	0.003
Non-union (%) (*n* = 2051)	11.3	12.5	11.0	0.688
Amputation (%) (*n* = 2079)	5.2	5.1	1.7	0.374
Follow-up (months)† (*n* = 1931)	10 (14)	14 (18)	9 (14)	< 0.001

Values are *mean(s.d.) and †median (range). A total of 2131 tibial open fractures are included in the analysis. ‡Pearson’s Chi-square test was used for comparing categorical variables and Kruskal-Wallis test for comparing continuous variables.

## Discussion

Moderate interobserver reliability has been reported for the Gustilo classification^10^, but it was unexpected to find that one-third of open tibial fractures requiring soft tissue reconstruction were classified as Gustilo I, II, and IIIA fractures initially. This proportion was higher in countries with no guidelines. Patients treated in high-income countries with established clinical guidelines had better access to healthcare and outcomes. Under-representation of studies conducted in less economically developed settings poses the risk of skewing data towards practices in higher-income nations. Guidelines for the management of open lower limb fractures advocate a multidisciplinary approach and global partnerships may offer quality improvement initiatives^11–13^. Patients treated in middle- and low-income settings experienced delays in accessing treatment, which was rarely delivered by multidisciplinary teams. Considering that lifelong limb prosthesis is costly, a tendency towards limb preservation in resource-constrained settings is expected^14^. There are inherent challenges associated with a methodology that relies on patient records in some institutions, including selection, information, detection, and collection biases. A prospective study would provide more reliable data that could include mental health and quality-of-life measures to refine global cooperative plans.

## Supplementary Material

znac105_Supplementary_Data

## Collaborators

Other members of the INTELLECT Collaborative: G. Nolan, H. Samarendra, A. Mohan, K. Cooper (Imperial College Healthcare NHS Trust, London, UK); N. Pereira, G. Mangelsdorff, J. Venegas, A. Layseca (Hospital del Trabajador de Santiago, Santiago, Chile); J. Skillman, A. Kennedy, A. Qureshi, K. Wallis (University Hospitals Coventry and Warwickshire NHS Trust, Coventry, UK); L. Harry, A. Hagiga, S. Ibrahim, M. Albendary, K. A. Shah (Brighton and Sussex University Hospitals NHS Trust, Brighton, UK); C. B. Chuo, C. Katsura (Hull University Teaching Hospital NHS Trust, Hull, UK); J. R. Rodríguez Astudillo, A. López Ortega, J. P. Henríquez Rissios, M. Nova Nova (Hospital Sótero del Río, Santiago, Chile); J. Hughes, C. Wearn, D. Peberdy, B. Ho, K. Gohil (University Hospitals Plymouth NHS Trust, Plymouth, UK); A. Abood, N. Rabey, M. Nizamoglu, G. Biosse-Duplan, K. To (Cambridge University Hospitals NHS Foundation Trust, Cambridge, UK); S. R. Sabapathy, M. Mohan, H. Venkatramani, S. Rajasekaran (Ganga Hospital, Coimbatore, India); H. Hsu (Dalin Tzu Chi Hospital - Buddhist Tzu Chi Medical Foundation, Dalin, Taiwan); A. R. Ambriz Plascencia, L. E. Escalona Ramírez, C. A. Zepeda Torres (Hospital Civil de Guadalajara, Guadalajara, Mexico); E. Santamaria, S. Vallejo Toro (Hospital General ‘Dr. Manuel Gea González’, Mexico City, Mexico); C. West, W. Bhat, C. McArdle, S. Louette, S. Hassan (Leeds Teaching Hospitals NHS Foundation Trust, Leeds, UK); P. W. van Egmond, W. J. J. Bekkers (Elisabeth-TweeSteden Ziekenhuis, Tilburg, Netherlands); D. Capitani, L. Troisi, T. Talamonti, P. Capitani, V. Cerbone, G. Materazzi, L. Ballini (ASST Grande Ospedale Metropolitano Niguarda, Milan, Italy); J. Tomas-Hernandez, J. A. Porcel-Vazquez, Y. Garcia-Sanchez, J. V. Andrés-Peiró, J. Teixidor-Serra, J. Selga-Marsà (Vall d'Hebron Barcelona Hospital Campus, Barcelona, Spain); H. Dafydd, S. Ali, R. Slade, S. Tarassoli (Morriston Hospital - Swansea Bay University Health Board, Swansea, UK); B. Olías López, J. Boluda Mengod, D. González Martín (Hospital Universitario de Canarias, Santa Cruz de Tenerife, Spain); A. Bashir, A. Dearden, V. Itte, F. Smith, C. W. Lee (The Newcastle upon Tyne Hospitals NHS Foundation Trust, Newcastle, UK); V. A. A. Paulus, P. Romijn, T. N. Tromp, T. de Jong (Radboud UMC, Nijmegen, Netherlands); S. Koide, K. Lim, F. Raiola, S. Ferris (The Alfred Hospital, Melbourne, Australia); A. Rodríguez, E. L. Jonsson, S. Holm, O. Wolff (Uppsala University Hospital, Uppsala, Sweden); A. Abugarja, H. Elbahari, H. K. S. Hamid, M. Awadelkarim (Ribat University Hospital and Ibrahim Malik Teaching Hospital, Khartoum, Sudan); J. Erdocia Pascual, L. Bahillo O'Mahoney, M. A. Quiroga Bilbao, M. Felipe Peña (Complejo Hospitalario Universitario Insular Materno Infantil, Las Palmas de Gran Canaria, Spain); W. Eardley, A. Egglestone, S. Taher, N. Wei (South Tees Hospitals NHS Foundation Trust, Middlesbrough, UK); J. Martínez Ros, G. Valero Cifuentes, A. Ondoño Navarro, A. Escudero Martínez, A. Ortega Columbrans (Hospital Clínico Universitario ‘Virgen de la Arrixaca’, Murcia, Spain); P. Zamora, J. Masiá, A. Ibarra, M. Fernández (Hospital de la Santa Creu i Sant Pau, Barcelona, Spain); V. Giblin, A. Kilshaw, B. Wood, M. Wyman (Sheffield Teaching Hospitals NHS Foundation Trust, Sheffield, UK), I. E. Tinhofer, E. Seidl, C. J. Tzou (Hospital of Divine Savior, Vienna, Austria); S. Quadlbauer, J. Reichetseder, H. Bürger, T. Hausner (AUVA Traumazentrum, Vienna, Austria); S. van Miltenburg, I. Beijk, W. Verra, R. de Groot (Medisch Spectrum Twente, Enschede, Netherlands); V. Kunc, L. Kopp (Masaryk's Hospital, Ústí nad Labem, Czechia); A. Crick, C. Mitchell, T. Curran, R. Kuo, S. Eltoum Elamin (Salisbury NHS Foundation Trust, Salisbury, UK); P. Caba Doussoux, D. Alonso Tejero, J. Gómez Alcaraz, J. M. Pardo García (Hospital 12 de Octubre, Madrid, Spain); K. Kooi, R. Poelstra (Medisch Centrum Leeuwarden, Leeuwarden, Netherlands); J. P. Hong, M. Jang, D. W. Hong, J. G. Kwon (Asan Medical Center, Seoul, South Korea); M. Francés Monasterio, J. Fernández-Palacios Martínez, A. Suarez Cabañas, M. Marrero Martínez-Carlón (Hospital Universitario de Gran Canaria ‘Doctor Negrín’, Las Palmas de Gran Canaria, Spain); W. ten Cate, J. E. D. Jacobs (Ziekenhuisgroep Twente, Hengelo, Netherlands); J. Rawlins, J. Haley (Royal Perth Hospital, Perth, Australia); J. Palma, A. Cuadra, H. Demandes, S. Canahuate, D. Moreno (Hospital Clínico Pontificia Universidad Católica de Chile, Santiago, Chile); S. Norton, J. Thompson, G. Lafford (Norfolk and Norwich University Hospitals NHS Foundation Trust, Norwich, UK); D. Noriego Muñoz, A. Teixido de la Cruz, M. Vázquez Gómez (Hospital Universitari Josep Trueta, Girona, Spain); A. Mingoli, D. Ribuffo, G. Marruzzo, P. Lapolla (Policlinico Umberto I - Sapienza University of Rome, Rome, Italy); W. Ayad, A. Elbatawy, M. Ouf (Al Azhar Hospital, Cairo, Egypt); P. Castillón, C. García, M. Surroca (Hospital Universitari Mútua de Terrassa, Barcelona, Spain); J. Garcia-Coiradas, F. Marco (Hospital Clínico San Carlos, Madrid, Spain); M. Cherubino, L. Garutti (Universita degli studi dell'Insubria, Varese, Italy); G. Molina Olivella, A. Endemaño Lucio (Hospital Consorci Sanitari Moises Broggi, Barcelona, Spain); K. Oflazoglu, F. Lutgendorff, M. Botman, G. Giannakopoulos (Amsterdam UMC, Amsterdam, Netherlands); R. Dams, P. van der Zwaal (Haaglanden Medisch Centrum, The Hague, Netherlands); R. Moral-Nestares, F. Requena (Hospital Universitario San Cecilio, Granada, Spain); N. Fernández-Poch, J. Cámara-Cabrera (Hospital Parc Tauli, Barcelona, Spain); F. Macán, M. Standen (Mutual de Seguridad, Santiago, Chile); F. Flaherty, M Vizcay (Sanatorio Otamendi, Buenos Aires, Argentina); D. Yerson (Sanatorio de la Trinidad, Buenos Aires, Argentina); E. Sperone (Sanatorio Finochietto, Buenos Aires, Argentina); F. Bidolegui, S. Pereyra (Sanatorio Sirio Libanés, Buenos Aires, Argentina); E. Chouhy, M. Jaureguialzo (Sanatorio Dupuytren, Buenos Aires, Argentina); Z. Arnez, V. Cazzato (Ospedale di Cattinara, Trieste, Italy); M. A. Giraldez, R. Moreno Domínguez, B. Martínez Sañudo (Hospital Universitario Vírgen del Rocío, Sevilla, Spain); L. Lancerotto, S. Sandhu (NHS Lothian, Edinburgh, UK); A. Robinson, C. Digney (Ulster Hospital - South Eastern Health and Social Care Trust, Dundonald, UK)

*Disclosure*. The authors declare no conflict of interest.
