# Supplementary material for: International Lower Limb Collaborative (INTELLECT) study: a multicentre, international retrospective audit of lower extremity open fractures
Source: Br J Surg. 2022 Apr 26;109(9):792–5. doi: 10.1093/bjs/znac105 (PMC10364752; doi:10.1093/bjs/znac105)
Supplement: znac105_Supplementary_Data [file znac105_supplementary_data.zip › Supplementary_material.docx]

**Supplementary material**

**Appendix S1: Methodology**

The International Lower Limb Collaborative (INTELLECT) study was a STROBE-compliant, international, multi-centre, retrospective audit coordinated by the INTELLECT steering group, with the support of the Reconstructive Surgery Trials Network (RSTN) based in the United Kingdom.

Orthopaedic, trauma and plastic surgery clinicians in centres that treat open lower extremity fractures were invited to participate through the RSTN and International Confederation of Plastic Surgery Societies (ICOPLAST) channels. A collaborative model for conducting research was used, based on previous success with this approach within the RSTN. No research ethics approval was sought for centres in the United Kingdom as this was considered an audit as per NHS Health Research Authority guidance; each participating centre registered this project with their Audit Department. For international collaborating centres, research ethics approval was obtained to comply with local or national regulations where necessary before commencement.

Investigators were tasked to retrieve data for patients treated with open lower extremity injuries between the 1^st^ of January 2017 and the 31^st^ of December 2018 according to pre-defined inclusion and exclusion criteria (Supplementary Table 1). This information was collected using an established REDCap^19^ online data gathering platform, hosted at the University of Oxford.

Data collection was undertaken following the General Data Protection Regulation (GDPR). All data were de-identified and securely stored in REDCap over the course of the study. Demographic and clinical information was obtained from medical records, including time to operative interventions, the surgical team involved, method of fixation and soft tissue reconstruction. The time from injury to definitive wound closure was treated as a continuous and categorical variable (within 72 hours, 3-7 days , 8-14 days, 15-28 days or >28 days post-injury). Primary outcomes were soft tissue infection, deep infection, non-union and amputation. Secondary outcomes were median time to discharge and deep venous thrombosis cases.

For quality assurance, each collaborating centre was asked to validate 2% of their cases, which were cross examined by members of the steering team. Case records were exported to an encrypted Microsoft Excel (Microsoft Corporation. Washington, USA) spreadsheet. Missing data were quantified for each variable as well as for the whole study cohort. Missing values were not considered for further analysis.

Statistical analyses were performed using IBM SPSS Statistics (IBM. New York, USA) software. A Shapiro-Wilk test was performed to assess the normality of the data. Descriptive analyses were undertaken to obtain frequencies and measures of central tendency. Bivariate analyses were performed with the Pearson’s Chi-square tests for comparing categorical variables and Kruskal-Wallis test for comparing continuous variables. Post-hoc analysis was performed using Bonferroni correction for multiple comparisons. Multivariate analysis was undertaken using binary logistic regressions to calculate the impact of the timing of interventions on primary outcomes following open fractures. Odds ratios (OR) with 95% confidence intervals were calculated and a p-value <0.05 was considered statistically significant.

Table S1: Inclusion and exclusion criteria

| Inclusion | Exclusion |
| --- | --- |
| - Patients admitted following an open lower limb fracture - Admitted between 1^st^ January 2017 and 31^st^ December 2018 - Any age | - Patients assessed at the participating centre but whose definitive treatment was performed at a different unit - Open forefoot and isolated patella fractures |

Table S2: Differences in mechanism of injury for male and female patients

| **Mechanism of injury** | Male | Female | p-value | Total |
| --- | --- | --- | --- | --- |
| Road traffic accident | 58.2% | 39% | p<0.001 | 52.6% |
| Low-energy fall | 10.6% | 37.9% | p<0.001 | 18.7% |
| High-energy fall | 10.9% | 11.1% | p=0.92 | 11% |
| Sports injury | 4.9% | 8% | p=0.002 | 5.8% |
| Work-related injury | 5.7% | 1.4% | p<0.001 | 4.4% |
| Interpersonal violence | 7.3% | 1.4% | p<0.001 | 5.5% |
| Other | 2.4% | 1.2% | p=0.05 | 2% |

Table S3: Multivariate logistic regression for days to obtaining wound closure

| Days to soft tissue cover | Wound infection | | Deep tissue infection | | Non-union | | Amputation | |
| --- | --- | --- | --- | --- | --- | --- | --- | --- |
|  | OR | p value | OR | p value | OR | p value | OR | p-value |
| 0-3 days | 1 | - | 1 | - | 1 | - | 1 | - |
| 4-7 days | 1.02 | 0.92 | 2.61* | 0.001 | 2.78* | <0.001 | 2.49* | p=0.12 |
| 8-14 days | 0.85 | 0.56 | 4.09* | <0.001 | 2.26* | 0.007 | 6.48* | p=0.001 |
| 15-28 days | 1.2 | 0.32 | 1.99* | 0.047 | 0.84 | 0.65 | 11.75* | p<0.001 |
| > 28 days | 3.07* | <0.001 | 7.4* | <0.001 | 1.52 | 0.19 | 5.34* | p=0.006 |
