# Supplementary figures and images for: International Lower Limb Collaborative (INTELLECT) study: a multicentre, international retrospective audit of lower extremity open fractures
Source: Br J Surg. 2022 Apr 26;109(9):792–5. doi: 10.1093/bjs/znac105 (PMC10364752; doi:10.1093/bjs/znac105)

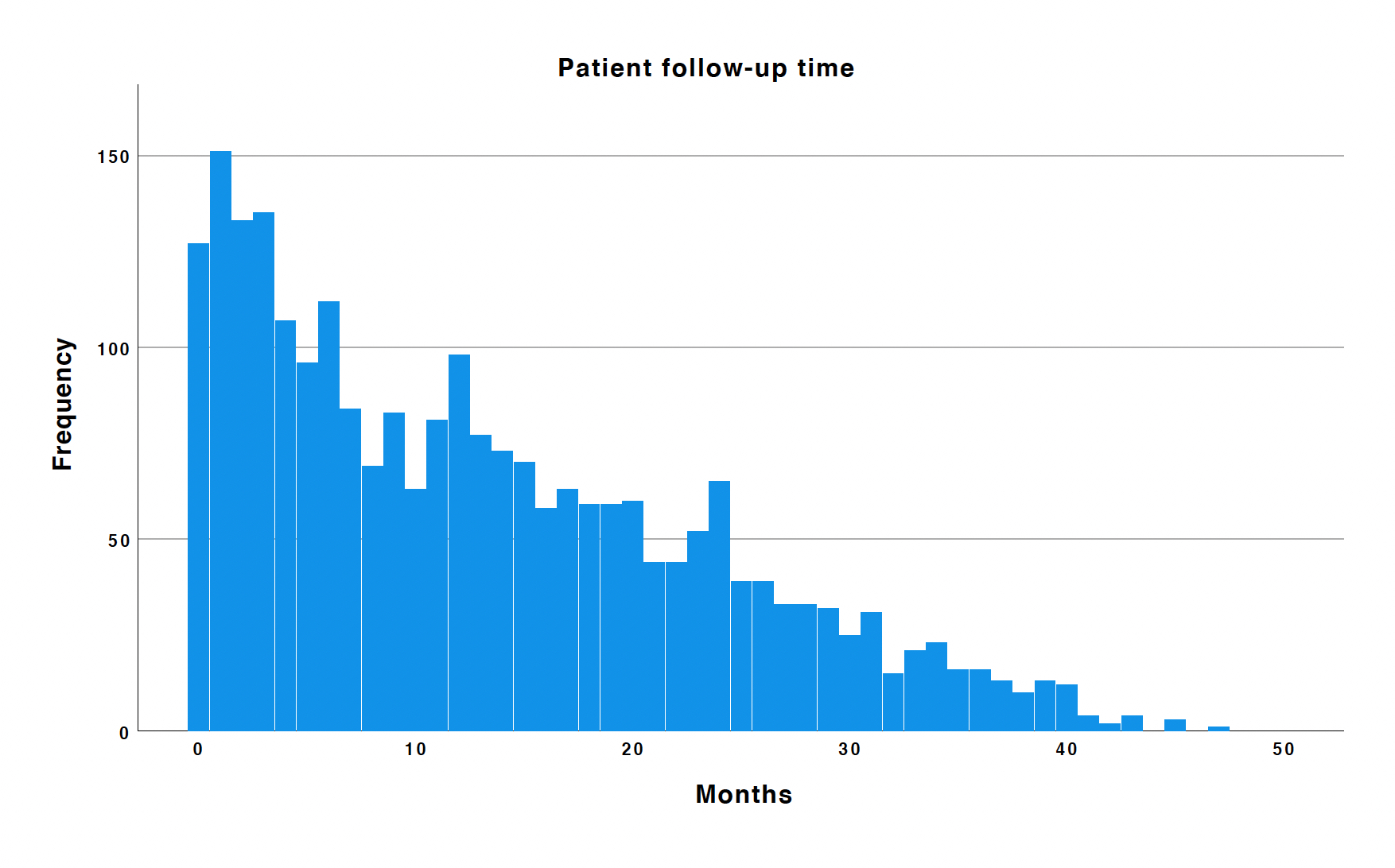

Supplement: znac105_Supplementary_Data [file znac105_supplementary_data.zip › Supplementary_Figure_1.jpg]
